# The History of Intravenous and Oral Rehydration and Maintenance Therapy of Cholera and Non-Cholera Dehydrating Diarrheas: A Deconstruction of Translational Medicine: From Bench to Bedside?

**DOI:** 10.3390/tropicalmed7030050

**Published:** 2022-03-12

**Authors:** David R. Nalin

**Affiliations:** Center for Immunology and Microbial Diseases, Albany Medical College, Albany, NY 12208, USA; nalindavid@gmail.com

**Keywords:** cholera, non-cholera dehydrating diarrheas, translational medicine, history

## Abstract

The “bench to bedside” (BTB) paradigm of translational medicine (TM) assumes that medical progress emanates from basic science discoveries transforming clinical therapeutic models. However, a recent report found that most published medical research is false due, among other factors, to small samples, inherent bias and inappropriate statistical applications. Translation-blocking factors include the validity (or lack thereof) of the underlying pathophysiological constructs and related therapeutic paradigms and adherence to faulty traditional beliefs. Empirical discoveries have also led to major therapeutic advances, but scientific dogma has retrospectively retranslated these into the BTB paradigm. A review of the history of intravenous (I.V.) and oral therapy for cholera and NDDs illustrates some fallacies of the BTB model and highlights pitfalls blocking translational and transformative progress, and retro-translational factors, including programmatic modifications of therapeutic advances contradicting therapeutic paradigms and medical economic factors promoting more expensive and profitable medical applications inaccessible to resource-limited environments.

## 1. Introduction

The intravenous (I.V.) and oral treatment of cholera and non-cholera dehydrating diarrheas (NDDs) provide insights into translational medicine, spanning the period from the birth of clinical laboratory science (the “bench”) in 1831 [1] to the development of modern oral rehydration and maintenance therapy (ORT) in 1967–1968 (the “bedside”) [2], which, in terms of saving lives, has been hailed as perhaps the most important translational advance of the last century [3].

A translational medical advance rests upon three foundations: a valid *causative* paradigm derived from a correct understanding of disease pathophysiology, a valid *therapeutic* paradigm for correcting the pathophysiologic disorder, and a clinically effective (and safe) *methodology* for delivering the treatment to provide a therapeutically beneficial or life-saving outcome. The ‘bench to bedside” slogan overlooks the fact that between bench and bedside are many factors which ultimately determine whether the therapy will be life saving, useless, impracticable, regressive or deadly. The notion that the bench to bedside concept of translational medicine proceeds in a linear manner is erroneous. Discoveries at the bench can take years and even decades to translate to widespread adoption and acceptance. In the case of intravenous (I.V.) and oral therapy (ORT) for cholera, the gap of 127 years from the first correct pathophysiologic and partial therapeutic paradigms for cholera (1831) to the 1960′s development of effective and safe treatment methods, and its application to NDD therapy, implies that a more nuanced understanding of transitional medicine is needed, based on historical review of the phases of translation.

## 2. Material and Methods

Cholera and the NDDs share major aspects of pathophysiology, and methods of rehydration and maintenance therapy for cholera are adaptable for treatment of NDDs. This review will examine the causes of the lethally slow progress toward an effective and safe cholera and NDD treatment regimen, including the pathophysiologic and therapeutic paradigms underlying NDD treatment from ancient times until the arrival of the first documented European cholera epidemic in 1830 (the apocryphal period); the years 1831–1947 (the transitional period); and 1948–1968 (the translational period). Numerous contextual translation-blocking factors and retro-translational factors delayed for 127 years translation to a safe and consistently effective treatment, factors which continue even today to play a negative role.

The key indicator of translational progress for cholera and NDD treatments is the case fatality rate (CFR). Historically, persistent high CFRs indicated translational paralysis. Treatments converging towards the modern CFR of less than 1% provide a key marker of translational progress, achieved by the timely replacement of diarrheal water and electrolyte losses with matching volumes of I.V. or absorbable oral solutions of similar electrolyte composition.

## 3. The Apocryphal Period

Before 1831, the true extent of cholera and its epidemic frequency remain unknown, due to lack of population-based clinical reports from the endemic areas in South Asia. Microbiology did not yet exist, and *Vibrio cholerae* Pacini was as yet unknown, as was cholera itself as we define it today [4]. Even clear, accurate, virtually pathognomonic clinical case descriptions such as Latta’s in 1831–1832 (vide infra) are not found in surviving documents before 1831.

In contrast, records of treatment recommendations for diarrhea go back several millennia [5]. The causes are today known to include a range from cholera and non-vibrio cholera (caused chiefly by various types of *Vibrio* sp. and *Escherichia coli*), and other potentially lethal NDDs of infants and children caused by rotavirus and other bacterial and viral pathogens.

While no quantitative or accurate CFR data exist before 1831, cholera CFRs of 40–60% or higher continued long after 1831 during a prolonged period of quantitative reporting up to the mid-20th century. Many preventable deaths still occur in cholera-affected areas lacking access to modern treatment. NDDs outnumber cholera in incidence and, before the development of modern therapeutic methods, took the lives of over five million under-5-year-olds annually [6,7].

During the “apocryphal” period from ancient times up to 1830, the main causative and pathophysiologic paradigms for diarrhea were (1) an imbalance of “humors” (bile, phlegm, blood, wind) attributed to Galen and Greek predecessors and (2) a poison in the blood, supposed to spread chiefly through the air [5]. Therapeutic recommendations were aimed at correcting imbalances of the humors or removing the poison by bloodletting and purging. Specific dietary or medicinal recommendations were not anchored in accurate therapeutic paradigms or proof of efficacy, but on alleged dietary or preventive practices of ancient hallowed authorities and/their modern interpreters, ranging from cereals or farinaceous gruels (Galen [8], p. 666) to raw oysters ([8], p. 677) (also Galen [8]). The treatments allegedly espoused by Galen and others over those centuries, including enemas to clean the intestinal surface and remove noxious intestinal contents ([8], p. 663), were as imaginative as the false theory of imbalanced “humors”. Though some of these reported recommendations such as gruel or soups of chicken or beef appear to echo modern clinical trial-based rice-, other cereal- or amino acid-based oral rehydration therapy [9], there is no more evidence for efficacy for raw oyster therapy than there is that gruels or soups of chicken or beef were tested in balance studies or administered in amounts sufficient to match voluminous fluid losses in accordance with any effective therapeutic method. Isolated uncontrolled case reports remain anecdotal, not translational.

Apocryphal claims of different foods, dietary and medicinal treatments for cholera became part of the material medica based on no quantitative evidence of clinical trials or objectively documented reports of reproducible successful clinical outcomes. Claims that recommendations for sugars, porridges, chicken soup and such represent early use of oral therapy for cholera or severe NDDs overlook the lack of any evidence that such dietary advice included any reproducible methods or reduced CFRs. A brief survey of such recommendations and linked therapeutic methods illustrates that many dietary recommendations are not equivalent to effective therapeutic methods. Use of a given food or solution without a linked effective methodology or proof of net absorption and concurrent reduction in CFRs is no translational breakthrough.

## 4. The Post-Apocryphal Transitional Period (1830–1959): Development of I.V. Therapy

Over more than a century after 1831, there is no mention of measuring and quantitatively replacing the diarrheal losses of water and electrolytes in a timely manner so as to avoid recurrent shock, renal failure and death. The method of bolus I.V. infusions given when shock recurred could not avoid decade after decade of 40 to 60% CFRs. Accurate pathophysiologic and therapeutic paradigms existed by 1831–1832, but in the absence of an effective and safe clinical methodology, the void was filled with a lethal array of irrational and often counterintuitive and contraindicated “remedies”.

Howard-Jones [10] has thoroughly reviewed the 19th c. advances in cholera pathophysiology and therapy and the numerous, mostly lethal traditional “treatments” of the time, most derived from ancient pathophysiologic paradigms based on irrational medical traditions supported by the medical hierarchy. Continued high CFRs confirm that successive treatment modalities failed to achieve life-saving outcomes during most of the 127 year evolution from bench to graveside rather than to bedside. Current misunderstandings of the progress, or lack thereof, in this period merit review of selected historical details.

By 1831, cholera had spread from Asia across Russia, Europe and into England, bringing mass panic and innumerable old and new “treatments”, often including contradictory modalities. The commonest remained bloodletting, the archaic causative and therapeutic paradigm being removal of an unknown toxic element in the blood. To this end, bloodletting and replacement with transfusion of human and even animal blood was attempted in the 1830s [11].

The arrival of cholera in Europe occurred at a time when the advances in chemistry and physics of the 18th century were first being applied to biomedical phenomena. Serum-specific gravity (sp.gr.) and chemical analyses of serum, clotted blood, cholera diarrhea fluid and vomitus were introduced, but the analytic skill of the pioneer clinical biochemists varied widely, and some specimens were analyzed fresh and others after long periods at room temperature.

The correct pathophysiologic and therapeutic cholera paradigms evolved from pathophysiological and therapeutic errors of William Stevens [12,13], who claimed cures using various salts to convert the color of blood of terminal cholera patients from cyanotic into a bright red color. In Moscow, Hermann published largely incorrect serum analyses but partially correct inference of loss of blood water (and, incorrectly, acetic acid [14]) in 1830. This led Jaehnichen to give a minute amount of I.V. water with acetic acid, apparently, from his published remarks, to lubricate the thickened and inspissated blood rather than to quantitatively replace the fluid losses of cholera. Then, the more accurate analyses, pathophysiologic and therapeutic paradigms of O’Shaughnessy inspired the daring clinical application of those findings of Latta (1831–1832 [15]), who advanced rational treatment as afar as possible in the absence of sterile solutions and a valid therapeutic method.

## 5. William Stevens’ “Saline Treatment”

The observations leading to the correct pathophysiologic and therapeutic paradigms for cholera were published in 1831–1832 during the European onslaught of cholera.

Stevens’ reported in 1830 [12] and again retrospectively in 1853 [13] that yellow fever patients had dark blood, indicating to him a lack of oxygen, and a bright red color returned on adding salt in vitro. This “saline method” was extended to the dark color of cholera patients’ blood as well, by giving small amounts of salt powder, saline solutions and saline and other laxatives by rectal enema, by steam bath, by mouth or injection (a word which in his texts is variably used to signify delivery not only parenterally in small amounts, but also by orointestinal or other routes). As noted by Howard-Jones [10], he had no concept of rehydration, only of inducing color change of the blood. He had no quantitative concept of cholera patients’ salt and water losses or of their quantitative I.V. replacement.

Modern balance studies have proven that oral plain saline is not absorbed by cholera patients and aggravates cholera diarrhea in the absence of glucose or other substrate in the solution to enhance active sodium transport and thereby also promote concurrent water absorption [16,17]. There is also evidence indicating the non-absorbability of colonic saline enemas during diarrhea [18,19].

A review of William Stevens’ claims in his book titled “Observations On the Nature and the Treatment of the Asiatic Cholera” [13] is warranted by the fact that despite his erroneous pathophysiologic and therapeutic paradigms and the non-absorbability in cholera patients of orally or rectally administered plain salt or saline solutions (or saline baths), several unwary authors [20,21,22] have in recent years mistakenly reported that Stevens was a pioneer in oral and I.V. rehydration therapy for cholera. This he was not, as critical reading of his publications demonstrates.

Stevens’ second book is a belated attempt to rescue his reputation and defend his claims of, in his words, “miraculous” cures of cholera patients using his “saline method”. However, Steven’s book gives no single clear or succinct description of his “saline method”. Several variants appear in different sections. In one, he states that “even at the eleventh hour, if a warm saline fluid be thrown into the intestinal canal, this vital fluid is even then rapidly absorbed by the absorbent vessels and the moment that this living fluid enters the circulation it gives new electric life to the blood.” ([13], p. lvii).

In contrast, Latta correctly reported that oral or rectal saline brought no benefit to cholera patients [15]. Stevens’ description of patients “throwing up” the oral salines are consistent with the lack of benefit of non-absorbable saline powders and laxatives. The latter promoted forceful evacuation along with the cholera stool, aggravating patients discomfort. Anal corks prevented expulsion of intestinal contents after laxatives and enemas [10].

Stevens held that “the non-purgative saline medicines were the most likely to be useful, for they not only redden the colour of the blood, but by increasing its fluidity they render it better fitted to serve its [intended].functions.” Applying this in Coldbath-Fields prison in 1832 to patients with presumed cholera with “premonitory symptoms of diarrhea and vomiting”, the treatment included a Seidlitz powder laxative, more active purgatives (e.g., sodium tartrate), Epsom salts added to the Seidlitz powder, and then, “on the bowels being moved, plenty of thin beef-tea, well seasoned with salt.” “If much irritability of the stomach prevailed, a sinapism (mustard plaster) was applied to the gastric region, and thirst was relieved with seltzer, soda or pure water ad libitum.” When the collapse stage was reached, a strong solution of the same salts, at a temperature of 100 degrees, was “thrown” into the bowels. Stevens claimed that the latter method succeeded far better than “injection of the vital electric salts into the veins” and patients “were generally dismissed cured in a few days.” [12,13]. Of these patients, none were entered as cholera cases in the prison journal, but Stevens attributed this to an aberration of prison recordkeeping.

There was no standard case definition of cholera, which was regarded as a fatal disease often, but not always accompanied with profuse diarrhea and vomiting, with stages based on the patient’s appearance and general condition: “premonitory”, later “collapse”, and (assuming survival after collapse) “reaction”. Case reports frequently included symptoms and signs not at all characteristic of cholera as we know it (some were even said to have constipation ([13], p. 39)). Stevens’ critics found his results unreproducible; many patients had mild diarrheas, or other non-diarrheal illnesses terminating in fatal “collapse” [23].

Stevens’ claims of “magical” cures were challenged by the medical authorities of the day, leading to the vituperations pervading much of Stevens’ 1853 book. These critics, including Sir David Barry, Mr. Wakefield and W.B. O’Shaughnessy, reported that examination of Steven’s reported cholera patients revealed that none of them had cholera. Stevens retorted that his critics “were the mere instruments in the hands of the higher human serpents, or the so-called physiologists, who are the leaders of the medical profession; for even to this day they are the false teachers that come in the garb of sheep’s clothing, but inwardly they are ravening wolves.” ([13], pp. 258–259).

An *enema (italics mine)* “composed of a large tablespoonful of muriate of soda dissolved in warm water, sometimes with the addition of sugar or starch” administered “at as high a temperature as the patient could well bear” was recommended, with additional “sinapisms….frictions.., warm towels” and “a pure air for the patient to breathe.”([13], p. 40).

Another patient received a Seidlitz powder laxative and mustard poultices with an “injection” (evidently per os, quotation marks mine) of four salines in a pint and a half of warm water, repeated until followed by a “copious motion” and then the saline powders were repeated every hour and saline injections used every three hours with warm flannel frictions and the third of a Seidlitz laxative given every half hour, and soda and seltzer-water …from time to time” ([13], p. 243). Then, “a saline injection attempted to be infused into the blood, but the veins were so completely collapsed it did not succeed. The patient …could not swallow, but a warm saline fluid was from time to time thrown slowly into the intestinal canal” ([13], p. 244]. The patient reportedly survived ([13], pp. 242–245).

In his 1832 book ([12], p.33), Stevens reported that 29 of 30 cholera patients survived after drinking 2 tablespoons of salt in 6 ounces of water hourly and one tablespoonful of a similar mixture cold every hour afterwards. Another iteration of the “saline method” as noted by Jones ([10], p. 386) included oral administration of “strong solutions of non-purgative neutral salts in half a tumbler of water” (see also ([13], p. 41)). Steven’s report has sufficient detail to establish that his “saline method” could not have had any therapeutic benefit due to the non-absorbability of plain oral saline or saline mixtures given by rectal or dermal routes. The variable components and unspecified quantities of other oral fluids and the tiny amounts given I.V., typically as a last desperate measure, could not avoid fatal outcomes. There was no awareness of the need, nor any method used to match volume of saline infusion by any route to volume of fluid losses.

In summary, there is no basis for considering Stevens an I.V. or oral rehydration or maintenance pioneer, as his salines, whether powder or liquid, oral drink, enema or bath, were non-absorbable in cholera and intended only to turn cyanotic blood red. He did not conceive of rehydration and conducted no measures of intake and output. The oral plain saline and saline laxatives that he recommended would have aggravated cholera patients’ diarrhea and dehydration. Use of oral salines in patients he claimed had cholera but who actually had other diseases may have been harmless or even beneficial in isolated cases, but provides no basis for considering Stevens as a rehydration pioneer. His “magical” cholera cures were evidently delusional and he confused matters further by hailing others’ correct concepts of intravenous saline replacement of the large volumes of fluid losses of cholera as if they represented proof and acceptance of his color change therapeutic paradigm ([13], p. 488–491). The controversies arising over the rejection of his claims by his contemporaries and his virulent condemnations of O’Shaughnessy and others who debunked his claims make it clear that his saline method falls into the category of ineffective cholera remedies of the period, lacking valid case definitions, correct pathophysiologic or therapeutic paradigms or effective clinical methods. The concept of controlled trials did not yet exist.

## 6. Rudolf Hermann and Friedrich Jaehnichen

Cholera’s progress through Russia led to medical commissions tasked with formulating quarantine and other supposed control measures. In Moscow, the German expatriate Hermann, a chemist, reported among the earliest measurements of serum sp.gr. in cholera and of other substances [14] which his faulty analyses convinced him were present in dehydrated cholera patients.

Hermann correctly interpreted the elevated serum sp.gr. as indicating loss of blood water, but his erroneous acetic acid measurements on cholera stools led Jaehnichen to conclude that I.V. water with added acetic acid might correct the inspissation of blood into clotted material blocking circulatory function along with the alkaline blood and acid stool erroneously identified by Hermann in cholera patients. Hermann performed no serum sodium, chloride or bicarbonate analyses, reporting only his erroneous finding of acetic acid, so Jaehnichen gave only 33 cc of dilute acetic acid (not saline or water) I.V. to relieve the thickening of the blood. The patient died soon after [24].

As Howard-Jones noted [10], Hermann and Jaehnichen had no concept of volumetric replacement of the diarrheal or emesis fluid losses. Hermann concluded that “the liquids evacuated in cholera both by the stools and by vomiting form constituent parts of the blood, which is deconstituted by their disappearance…the immediate cause of death is consequently the thickening of the blood, which prevents its circulation.” ([10], pp. 385–386). He continued that this “decomposition” was due to the “separation of the acid and aqueous liquids that are evacuated in the diarrhea and vomitus of cholera patients.” ([14], p. 29).

Hermann surmised that intestinal absorption was blocked in cholera based on an alkaline diarrhea fluid of a cholera patient fed a sodium hydroxide solution and speculated that the diarrhea fluid originated as a transudate across the intestinal wall (later disproven).

Hermann ([14], pp. 29–30) agreed with Jaehnichen and others that cholera was an affliction of the symphatique or “pneumo-gastrique nerve”. Here again is an ironic and seemingly prescient but totally unrelated idea which in modern times has emerged as a role for the nervous system and gut hormones in choleragenesis [25]. The chance use of seemingly prescient but actually unrelated descriptive phrases often suggests ideas that the authors could not have understood as we do today.

All Hermann’s reported analyses except the elevated serum sp.gr. were erroneous, including absence of urea in anuric cholera patients. His finding of blood and stool acetic acid may have resulted from detecting indole-3-acetic acid, a product of bacterial metabolism found in contaminated blood and intestinal fecal matter [26]. Hermann noted that others had in ten cases found the stools to be alkaline, not acidic. This he attributed to “national differences” He also noted that other investigators at the time had reported highly elevated serum sp.gr. values in terminal cholera patients (e.g., Thompson: 1.057; Rose and Willstoch (Berlin): 1.0447 ([14], pp. 36,40). He believed that cholera blood had lost its normal anatomical shape and was “decomposed.”

Hermann and Jaehnichen may have been first to connect the pathophysiologic dots between raised serum sp.gr. and loss of water from blood via diarrhea and vomitus. However, their therapeutic paradigm focused on the idea that the desiccated thickened blood was decomposing into woody and polypoid masses which caused death by blocking the circulation, compromising the heart. Their therapeutic paradigm was inappropriately focused on eliminating the inspissation of the blood rather than replacement of the large fluid losses, the true composition and volume of which they had failed to identify.

Hermann was not a physician and uncritically recorded the many irrational medical practices then prevailing ([14], p. 33) and not rejected by him or Jaehnichen, such as venesection and commonly used ineffective and often lethal medicinals, herbal preparations, plasters, baths and enemas. These were essentially placed on a par with the idea of replacing cholera’s fluid losses with small intravenous injections. Hermann’s incorrect analyses and his omission of sodium and bicarbonate analyses led to Jaehnichen omitting the crucial electrolytes lost in cholera diarrhea. Had Jaehnichen realized the volumes that were needed, and had he administered them as dilute acetic acid, fatal massive hemolysis would have occurred.

## 7. O’Shaughnessy

In London, O’Shaughnessy at first pursued Stevens’ red color theory and advocated giving salts with more oxygen than sodium chloride to redden the blood ([27], p. 31, line 434). He read Hermann’s reports and, having performed his own remarkably accurate determinations of sp.gr., sodium, chloride and bicarbonate levels in cholera patients’ serum and diarrhea fluid, he published a critical report (10, p. 387 [28]) indicating correctly that all but Hermann’s finding of an elevated serum sp.gr. were in error, including the findings of acetic acid in the cholera diarrheal fluid, an alkaline serum and absence of urea in anuric cholera patients’ serum.

O’Shaughnessy noted that Hermann’s specimens were mishandled, resulting in decomposition with erroneous high levels of alkali in cholera patients’ blood and acetic acid in cholera diarrhea. Modern analyses confirm the opposite: acidosis in the blood and highly alkaline diarrheal fluid [29].

O’Shaughnessy’s new pathophysiologic paradigm, whether guided by (or in opposition to) Hermann’s and Jaehnichen’s reports or not, led him to the essentially different and correct pathophysiologic paradigm of loss of water, salt and bicarbonate causing the cholera syndrome, with the logical therapeutic paradigm being replacement of the water and salt losses with I.V. solutions. The need for aseptic technique and sterile infusions remained undiscovered translation-blocking factors.

O’Shaughnessy’s analyses led to these conclusions [28]:

1. The blood drawn in the worst cases of the cholera is unchanged in its anatomical or globular structure.
2. It has lost a large proportion of its water, 1000 parts of cholera serum having but the average of 860 parts of water.
3. It has lost also a great proportion of its neutral saline ingredients.
4. Of the free alkali contained in healthy serum, not a particle is present in some cholera cases, and barely a trace in others.
5. Urea exists in cases where suppression of urine has been a marked symptom.
6. All the salts deficient in the blood, especially the carbonate of soda, are present in large quantities in the peculiar white dejected matters.

As Howard-Jones noted [10], p. 387] O’Shaughnessy’s therapeutic recommendations were “(1st) To restore the blood to its natural specific gravity and (2nd) To restore its deficient saline matters. The first of these can only be effected by absorption, by imbibition, or by the injection of aqueous fluid into the veins. The same remarks, with sufficiently obvious modification apply to the second.” Absorption and imbibition were soon found blocked in cholera.

However, O’Shaughnessy continued to recommend “other remedies: such as stimulants, opioids, external warmth, etc., “which may be calculated to re-excite the circulation and promote the required absorption… and “tepid water enemas containing a certain proportion of the neutral salts” ([10], op cit; [28], p. 53). Unaware of the absorptive defects in cholera, he stated: *“I would expect much benefit from the frequently repeated use of the neutral salts by the mouth or by enemata*.”([10], op cit, p.-54) while noting that the salts in most cases “pre-exist in the intestinal canal.” His crucial correct inference was that: “In the severe cases in which absorption is totally suspended, and when stimulants…fail to re-excite the circulation, I would not hesitate to inject some ounces of warm water into the veins …and “dissolve in that water the mild innocuous salts …which in cholera are deficient.” His conjecture would have been more accurate had he said “pounds” instead of “ounces”

He proposed a formulation with 6 ounces of water, to be repeated every 2 h, containing sodium phosphate, sodium chloride, sodium carbonate and sodium sulphate, then covering his bets by “also obey[ing] every local indication and use cold applications, leeches, etc.”.

## 8. Latta

Inspired by O’Shaughnessy’s publications, Latta decided to replace the losses orally, rectally or I.V. with significant volumes of saline solutions and noted for the first time the failure of oral or rectal plain saline solutions to provide any benefit [15]. Treating cholera patients at an advanced stage of their dehydration, or “collapse”, he infused up to ten pounds of his saline solution using the Reid’s syringe used to bleed patients, and his vivid and accurate descriptions noted the immediate striking improvement in moribund cholera patients given such treatment. His solutions were hypotonic, containing less sodium than in cholera diarrhea ([15], pp. 208–213) but capable of reviving patients without inducing hemolysis.

Latta wrote ([15], pp. 275–277), “As soon as I learnt the result of Dr. O’Shaughnessy’s analysis, I attempted to restore the blood to its natural state, by injecting copiously into the larger intestine warm water, holding in solution the requisite salts, and also administered quantities from time to time by the mouth, treating that the power of absorption might not be altogether lost, but by these means produced, in no case, any permanent benefit, but, on the contrary, I though the tormina, vomiting and purging were much aggravated thereby, to the further reduction of the little remaining strength of the patient; finding thus, that such, in common with all the ordinary means in use, was either useless or hurtful, I at length resorted to throw the fluid immediately into the circulation….. The first subject of experiment was an aged female, on whom all the usual remedies had been fully tried, without producing one good symptom; the disease, uninterrupted, holding steadily on its course, had apparently reached the last moments of her earthly existence, and now nothing could injure her—indeed, so entirely was she reduced, that I feared I would be unable to get my apparatus ready ere she expired. Having inserted a tube in the basilic vein, cautiously—anxiously, I watched the effects; ounce after ounce was injected, but no visible change was produced. Still persevering, I thought she began to breathe less laboriously, soon her sharpened features, and sunken eye, and fallen jaw, pale and cold, bearing the manifest impress of death’s signet, began to glow with returning animation; the pulse, which had long ceased, returned to the wrist; at first small and weak, by degrees it became more and more distinct, fuller, slower and firmer, and in the short space of half an hour, when six pints had been injected, she expressed in a firm voice that she was free from all uneasiness, actually became jocular and fancied all she needed was a little sleep; her extremities were warm, and every feature bore the aspect of comfort and health. This being my first case, I fancied my patient secure, and from my great need of a little repose, left her in charge of the hospital surgeon; but I had not been long gone, ere the vomiting and purging recurring soon reduced her to her former state of debility. I was not apprised of the event, and she sunk in five and a half hours after I left her…. I have no doubt the case would have issued in complete reaction, had the remedy, which already had produced such effect, been repeated.”

Writing to express his pleasure at hearing Latta’s results, O’Shaughnessy could not resist making some further therapeutic suggestions, including minute doses of astringents and stimulants (ammonia carbonate intravenously based on its toleration by horses, quinine sulfate, dilute spirits and weak herbal decoctions ([30], p. 281).

Refining Latta’s therapeutic recommendations, Lewins ([31], pp. 243–244) noted that “a large quantity must be injected, from five to ten pounds in an adult and repeated at longer or shorter intervals as the state of the pulse and other symptoms may indicate; whenever the pulse fails, more fluid ought to be thrown in to produce an effect…without regard to quantity.” However, no treatment omitting the quantities of the fluid losses or replacing all the losses as they occurred could lower CFRs.

Influenced by the prevailing concept of cholera as a disease with the three phases of premonitory, collapse and reaction, treatment began too late and was focused on correcting the collapse stage, which initial intravenous boluses appeared to do, after which Latta recommended astringent enemas, hoping to stop the diarrhea and prevent recurrent shock ([15], p. 276).

The concept of continuous maintenance therapy after correction of shock to prevent its recurrence, obvious in retrospect, had not occurred to anyone at this point. As Latta reported, the initial startling improvement following such I.V. infusions was usually followed by recurrences of collapse with frequent fatal termination. The actual average volumes of I.V. saline given, though sufficient to transiently restore blood pressure and pulse, were far below the amounts needed to replace the total volume of ongoing cholera fluid losses.

Two translation-blocking factors prevented Latta from achieving reliable recoveries and wider acceptance of his innovation. Though he recognized that the patients who died after initial I.V. saline infusions resuscitated them did so because of lack of timely repetition of the infusions, the lack of a therapeutic method ensuring effective and timely maintenance therapy after initial rehydration ensured continued high CFRs. Though serum sp.gr. was measurable, its use as a monitoring tool to guide therapy escaped notice and/or actual practice. Additionally, Latta’s sharp clinical eye, proven by his vivid descriptions, did not lead to a standard method of monitoring clinical signs (changes in degree of sunkenness of eyes, tenting of skin, etc.) to guide maintenance therapy. Without a means to monitor ongoing losses and replace them in a timely manner, severely ill cholera patients invariably faced multiple episodes of recurrent dehydration and shock, most ending fatally.

Though Latta described using “warm” I.V. saline infusions, the problem of the late often fatal “febrile stage” indicates that the solutions and implements used were not accidentally sterilized as might have happened had he first boiled the water, then allowing it to cool to the desired “warm” temperature.

Regardless of the lethality of repeated rehydration rather than maintenance therapy after rehydration, medical microbiology remained undiscovered, leading to a “typhoidal” stage of fatal sepsis after I.V. injections of unsterile solutions. Lethal air emboli were another impediment to wide acceptance [10].

Secondary factors which contributed to the stagnation of Latta’s I.V. saline therapy for cholera were his death in 1833, the waning of the 1831–1833 cholera epidemic in the U.K. and Europe and, when it returned, the continued popularity among physicians of bleeding and myriad ineffective, often harmful traditional remedies and procedures. Practitioners trying I.V. saline infusions reported mixed, usually transient results ([31], pp. 292–293) followed by deaths. CFRs were acutely typically 70% or more; no method of measuring input and output was developed. Controversy over Latta’s treatment preceded his death from tuberculosis in 1833 [32].

Another less recognized factor was the “tinkering effect”, or the misguided modifications of formula or methods by various medical authorities introducing inadequately evaluated innovations, whether useless, harmful or beneficial. For example, the use of I.V. albumin by Parkes ([10], p. 391) and others or of I.V. milk infusions by Bovell ([10], p. 394) had disastrous effects, helping to discredit the method.

I.V. saline was tried in Madras Presidency in 1832 but abandoned, although transient improvement was reported [32]. O’Shaughnessy went to India in 1833, where he helped introduce the telegraph and practiced an early form of photography ([33]. Though a medical officer, no evidence exists that he continued cholera studies or altered the beliefs or practices of colonial administrators or physicians during the annual cholera epidemics, though Macgregor in 1838 in South India indicated he would use Mackintosh’s version of I.V. therapy (vide infra) in “bad” cases [32]. An interesting example of post-Latta practice was the work of William Marsden, who published a summary of his cholera treatments from 1832 to 1834, with follow-up editions from 1848 to 1865 after cholera recurrences [34].

He notes O’Shaughnessy’s publications and, while ignoring Latta’s, espouses I.V. saline infusions for advanced-stage cholera. He noted patients’ extreme thirst, but proscribed oral fluids, fearing vomiting, while continuing to recommend Stevens’ “saline” treatment, water (to clean the bowels) and also the purgatives and 2 h hot baths containing 7–14 pounds of salt for relief of cramps. He shared the opinion of those suggesting a (mesenteric) neuronal mechanism of cholera. Marsden recommended Stevens’ oral salt powders dissolved in a small quantity of water q 15 min (p. 46) to suppress the disease in the first stage (before collapse) and larger saline solutions (followed by water) to clean the intestines. If the patient remained pulseless, he followed O’Shaughnessy’s recommendation of “fluid corresponding in character as nearly as possible to the serum of the blood, injected in sufficient quantity to fully restore the pulse”. Afterwards, opium (otherwise condemned) and quinine, and then for survivors, rice, other farinaceous puddings and malt liquor. A subsequent septic “typhoid” stage was again noted. His CFRs ranged from 31 to 90% in patients receiving various treatments ([34], p. 61). One series with remarkably high survival rates clearly included only patients with non-cholera diarrheas.

Like Latta, Marsden gave I.V. saline chiefly to terminal cholera patients after pulse disappeared, noting that attending physicians must not be away for over two hours to avoid losing patients due to unattended relapses; but his CFRs indicate that such absences were the rule rather than the exception.

Marsden’s use of rectal cannulas for I.V. infusions is notable in the days before autoclaving. Sepsis due to unsterile infusions and a faulty method of replacing the large unmeasured ongoing losses, by waiting for shock to recur, remained key translational blocks to widespread adoption of I.V. saline as the treatment of choice. The same faulty therapeutic methodology would ensure high CFRs until after 1959 [29].

In the decades after Marsden, numerous practitioners tried various I.V. and oral treatments, among them MacIntosh, Magendie, Broussais, Lizard, and Wall. MacIntosh, in 1836, used an I.V. solution close to normal saline and, like Latta, added bicarbonate. An experimental addition of I.V. egg albumen was quickly abandoned. CFR was 84% ([10], p. 391) but this was attributed to tardy treatment, the ill-fated but often repeated practice recognized by Latta. Magendie’s cure-all “punch” contained a pint of chamomile infusion, 2 oz. of alcohol, 1 oz. of sugar and lemon juice, along with frictions and heat. No method of administration was indicated; 27% survival was claimed [35]. Broussais [36], like many others, advocated what he called his “physiologic method”, including vapor baths, leeches, plasters and sweet drinks, the latter given to induce vomiting rather than rehydrate. He reported 97.5% survival using these treatments, but the Gazette Medical reported only 17%. Lizard [37], following Delpech, reported success in 30 cases with intravenous saline and oral alkali. In 1893, Wall used an I.V. solution similar to MacIntosh’s but with more bicarbonate (24 mEq/L.) [38], and added gelatin. CFR was 70% [39]. Lewis [40] used oral alkali.

Borne by trade and the burgeoning shipping industry linking endemic to non-endemic areas, cholera revisited England and the continent repeatedly during the 19th c., with Latta and Lewins’ urging of persistent fluid replacement being ignored, little or no effective treatment and a resurgence of maniacal or quack “cures”: extract of lamb testicle can be added to Jones’ tally [41], along with a costly venerable concoction attributed to Galen, called Theraica, consisting of wine, spices, purgatives and viper’s broth [42]. Stevens’ blood color-changing “salines” continued to be debunked ([43], p. 64–65). Amid this morass, Pacini, who discovered cholera vibrio, also advocated I.V. saline replacement therapy [44] using a formula close to normal saline. Sterilization by boiling of water to prepare solutions for I.V. use gained usage by 1892 ([45], p. 151) though pyrogens, while not usually fatal, were not removed until 1938 ([46], p. 784).

The medical establishment continued to ignore or reject sound therapeutic paradigms; and medical textbooks such as Osler’s ([47] p. 232–233) in 1907 advocated apocryphal treatments such as morphine, reduced or enhanced oral intake (ice, brandy or coffee and other drinks), cocaine, hot water lavage, heat, hot baths, hypodermic injections of ether and enteroclysis with warm water and soap or tannic acid (3–4 L), as in the 1902 edition [10]. The sign that the enteroclysis had been thoroughly accomplished was when the patient vomited the tannic acid bowel irrigant. S.C. saline infusions (4 g/L) and I.V. milk were recommended “until the pulse returned”. Relying on return of a failing pulse to guide repeat infusions [48] failed to save patients, as in Lewins’ day.

## 9. 20th Century: Rogers

Sellards’ advocacy of bicarbonate or base precursors [38,49], used eighty years earlier by Latta [15], led Rogers to add alkali to his hypertonic saline regimen, though Sellards’ advocacy was based chiefly on the mistaken idea that the alkali would prevent uremia. When Osler’s text was updated by McCrae [50], revised recommendations included alkaline stomach washes, hot baths, abdominal heat, castor oil or calomel purgatives, opium, kaolin, potassium permanganate and pituitary extract and caffeine in case of collapse, and Rogers’ hypertonic saline injections (I.V., rectal, intraperitoneal (I.P.) or subcutaneous (S.C.)) [51,52], repeated as needed to keep the blood pressure above (a mere) 70 and the sp.gr. below 1.063. If uremic, the saline plus bicarbonate solution was recommended.

Rogers saw the gut as a passive osmotic membrane, assuming that I.V. hypertonic saline would draw the diarrheal fluid back into the blood, preventing death [51]. This in fact did not occur, nor did it account for potassium or bicarbonate losses or for adverse effects of large infusions of hypertonic saline. Serum sp.gr. was used to estimate initial degree of dehydration and rehydration fluid needs but was not consistently used to monitor hydration status continuously to match I.V. fluid volumes to losses to reduce CFRs; sp.gr. was eventually omitted from Rogers’ cholera treatment recommendations [53]. Fixed amounts (4 pints) of I.V. hypertonic saline were given during the collapse or “algid” stage, then stopped [53]. CFRs rarely fell below 30% [52], far above those ultimately achievable with further advances in replacement solutions and therapeutic methodology.

Rogers’ methods were becoming more critically viewed, and modern methods of analysis were reapplied to studies of cholera pathophysiology. Recommendations from the British War Office as late as 1946 [53] noted that drugs were chiefly of little use, but the traditional castor oil purgative (with brandy) and morphine for vomiting were retained. Oral glucose was permitted, with Coramine, kaolin and pituitary extract for persistent hypotension. Rogers’ I.V. saline with bicarbonate (and rectal saline) were noted, guided by blood sp.gr. and blood pressure, but blood pressure monitoring replaced sp.gr. for faster mass treatment, and the need for hypertonic saline and alkali solutions was questioned; normal saline being simpler to formulate and more rapidly administered. I.V. glucose (10–25%) or 4.5% S.C. without rationale was strongly advocated, However, archaica such as essential oils, mixtures (opium, bismuth salicylate, chloroform, etc.) and rectal tannin enemas, poultices and dry cupping over the kidneys, persisted, and waiting for blood pressure to fall risked fatalities if used as the sole monitoring method without intake and output measurements.

## 10. Robert A. Phillips

Phillips and co-workers in the U.S. Navy clarified the composition of cholera’s “rice water” diarrhea and provided guidelines for appropriate I.V. replacement solutions. Applying the balance study technique, they developed optimal methods for rehydration and maintenance of patients with severe dehydration due to cholera and other severe NDDs.

Phillips had devoted his professional life to research on cholera and its effects on intestinal absorption of water and electrolytes. In 1948 [54], he and coworkers used modern analytic methods to precisely compare the electrolyte content of cholera patients’ blood and “rice water” diarrhea. They used I.V. normal saline with added bicarbonate and potassium to replace the water and electrolyte losses [55]. However, their method of therapy was based on bladder catheters to monitor urine output; and after initial rehydration, they gave maintenance fluids only when urine output fell after severe dehydration recurred, too late to totally eliminate fatalities. Though Phillips had developed the copper sulfate method of measuring blood sp.gr. (other solutions had been used earlier), it was not used in Egypt to closely monitor hydration status, nor were diarrhea fluid volumes measured. Patients were at risk of recurrent severe dehydration, and 3 of 40 patients died [55].

Contemporaneously, several authors, perhaps inspired by parallel pediatric recommendations, suggested calf or human [56] plasma infusions for cholera. Total I.V. requirements were estimated from initial serum or plasma sp.gr. or monitored by venous pressure. Both oral and I.V. glucose with half-strength saline were recommended by Chaudhuri [57], equally with oral plain saline, water or other beverages.

Continuing his pursuit of better treatments at NAMRU II in Taipei with Watten and colleagues, the method of measuring the actual volumes of diarrhea and vomitus of cholera patients (along with plasma sp.gr. monitoring) and replacing them with matching volumes of I.V. fluids containing appropriate electrolyte content was developed further. As in the earlier Egyptian studies, they used chiefly normal saline, later adding sodium bicarbonate and potassium chloride as needed to correct acidosis and hypokalemia [29]. This method could eliminate fatalities if delivered in a timely manner, with I.V. rehydration followed by I.V. maintenance therapy given continuously to match diarrhea and vomitus losses and avoid recurrent dehydration, shock and renal failure. Monitoring clinical signs could obviate plasma sp.gr. measurements for routine clinical use [58,59,60].

A wood-frame (“Watten”) cot with a covering plastic sheet with a sleeve entering a calibrated translucent bucket or one with a dip stick below [59] facilitated both balance studies and management of cholera epidemics, and could be mass manufactured on short notice. Physicians or nurses could monitor the level of diarrheal fluid lost q4–6 h by glancing at the bucket and checking that the volume of I.V. fluids matched volume of losses.

Later, at the Pakistan-SEATO Cholera Research Hospital (PSCRL) in Dacca (now the International Center for Diarrheal Diseases Research, Bangladesh, or ICDDRB, Dhaka, Bangladesh), Robert Gordon and colleagues introduced a single I.V. solution matching the electrolyte composition of adult cholera patients [60], and usable in pediatric cholera patients, whose rice water diarrhea contained moderately less sodium chloride and more potassium. This original “Dacca Solution” contained bicarbonate, but later versions replaced non-sterile B.P. grade sodium bicarbonate (requiring special equipment to sterilize) with a base precursor such as acetate [61] or citrate [62], both ingredients simpler to autoclave. Lactate also had adherents [58] but etched glass I.V. bottles. Oral citrate had been used earlier in an incomplete formula [63].

## 11. Parenteral to Oral Therapy: From Glucose for Calories to Glucose–Sodium Coupled Transport

The translational mishaps that marked the historical path toward an I.V. treatment and method reducing cholera CFRs virtually to zero were paralleled by a similarly complicated series of events and non-events characterizing the development of a safe and effective oral therapy for cholera and NDDs [6,7].

The Lancet statement that “The discovery that…glucose accelerates absorption of solute and water…was potentially the most important medical advance this century” [3] focuses on the scientific advances in understanding of intestinal absorption mechanisms, particularly the co-transport of glucose and sodium ions, enhancing salt and water absorption. The statement reinforces the conventional translational paradigm of bench to bedside, based on the assumption that the in vitro or animal model studies starting from 1959 [64] linking sugar and salt absorption led directly to the demonstration of an oral therapeutic paradigm and method capable of reducing [2,65] or eliminating [66,67] the requirement for I.V. therapy and were the sine qua non of oral therapy’s translational success.

In fact, the historical sequence illustrates that empirical clinical observations rather than basic science discoveries led to new pathophysiologic and therapeutic paradigms. Key insights into therapeutic methodology then rescued advances from failures. The prevailing wisdom that oral intake would aggravate diarrhea during the acute phase of illness had to be overcome by the marriage of empirical clinical research and post facto confirmatory basic science to establish that while the accurate therapeutic paradigm remained essential, the key to translational success was not the route of delivery per se, but the absorbability of the delivered fluid and an effective therapeutic methodology ensuring net positive gut balance, i.e., that intake exceeded output.

As early as 1824 in India, where British health workers almost universally advocated bleeding cholera patients and withholding all oral fluids, William Scot [68], considering the latter highly questionable, stated that “are we to pay no attention to the dreadful feeling of thirst, which forms so general and so distressing a symptom of the disease, and are we to disregard the state of the body, robbed, as it evidently is, in most instances, of all its serous and aqueous parts?...” The free use of diluents is indicated by the raging thirst, which prevails, and by the extent of the discharges, which evidently drain the system of a large portion of its serous or watery parts. He recommended tepid diluent fluids such as acidulated water, barley, rice, sago or arrow root decoction, chicken water or beef tea given freely from onset (wine or spirits to be diluted in arrowroot or sago). Rice water or pepper water could obviate caste restrictions on other liquids. Unfortunately, no methods or quantitative advice were included, and he proceeded with recommending the litany of opium, calomel, bleeding, and ipecac.

As noted above, Latta, in 1831–1832, had correctly reported [15] the failure of plain oral (and rectal) saline to benefit cholera patients; and while Stevens had reported its “miraculous” benefits, his work had been discredited, though some continued to believe it.

The first study linking salt with enhanced glucose absorption was published by Reid in 1901 [69] but led to no oral therapy breakthrough, because Reid focused on the increase in glucose absorption in the presence of salt, overlooking the obverse effect of glucose on salt and water absorption. Considering salt entering the lumen to be a sign of deterioration of his dog loop model, he curtailed his studies. There was apparently no awareness that this was the normal role of sodium and chloride in intestinal osmoregulation.

Neither Reid nor anyone in the medical world was studying cholera pathophysiology or new treatments; Rogers’ hypertonic saline was soon to become the accepted therapeutic modality. The prevailing colonial order had little or no concern about cholera in the native populations not served by any adequate medical facilities. Mortality from NDDs was very high in the U.S. and Europe at the time, but pediatric therapy was based on avoiding oral intake in the belief that oral intake would aggravate diarrhea during the acute phase of disease. Reid’s discovery is an example of how a potentially ground-breaking basic science observation published in a contextual vacuum can lead nowhere.

## 12. Hospital-Based Use of Oral Electrolyte Solutions with Glucose Added to Boost Caloric Intake

Up to the mid-20th C., pediatric diarrhea therapy included an initial period with no food given for 24 h and often longer. The treatment of cholera recommended by Osler in 1902 has been recorded by Howard-Jones [10]. By 1907, his treatment of pediatric NDDs included alkaline stomach washes with castor oil or calomel purgatives with opioids and chloroform, including laudanum enemas at six hourly intervals [47]—for alkaline stools (then regarded as a sign of protein decomposition), carbohydrates (barley water); and for acid stools, “beef juice”. Water and bicarbonate was delivered by mouth or bowel (for acidosis) and normal saline by the slowly absorbed S.C. route, which had highly undesirable effects including pain, infection and tissue damage.

As noted earlier, dietary recommendations per se, though perennially popular, are not equivalent to effective treatments, i.e., use of a given food or solution is not by itself, without a linked effective methodology and proof of maximal reduction in case fatality rates, a harbinger of later medical breakthroughs.

Brown and Boyd [70] in 1922 advocated 12–48 h starvation, allowing oral water, S.C., I.P. and I.V. saline and glucose, and exsanguinating transfusions to remove supposed toxins; CFRs were 39–80%. The ill-defined and inappropriate concept of “infantile intestinal intoxication” long persisted as a clinical misnomer.

Powers in 1926 [71] recommended blood transfusions and starvation up to 12 days. The rationale for blood or plasma transfusions was never clear, but was regarded as a way to correct “shock”, actually caused by severe water and electrolyte loss leading to severe dehydration, not by blood loss. Regarding oral fluids, he stated “By mouth… we give water; we have tried Ringer’s solution and 2 to 5% glucose both in water and in Ringer’s solution. We have been unable to observe, as yet, any advantage in any solution over water.” CFR was 33%.

The fundamentals of pediatric diarrhea therapy for decades remained the withholding of oral intake for a variable period [70,71,72,73,74,75,76,77,78,79,80,81,82,83,84,85,86,87,88,89,90,91,92]. Gamble in 1943 suggested up to 20 days without oral intake and some considered adequate oral intake sometimes impossible even without vomiting [72]. Administration of electrolyte solutions ***or*** glucose by I.V., slow S.C. or hazardous I.P. routes, along with plasma or whole blood transfusion, was considered standard therapy. Resumption of oral intake could begin after correction of dehydration using parenteral routes, but the lack of knowledge about the role of glucose in intestinal salt absorption led variously to recommendations for oral glucose, plain water, plain saline, half-strength Hartmann’s solution, glucose with saline or sucrose with saline **[73,76]**. Some of these recommendations were potentially harmful by augmenting diarrhea or sodium losses and by aggravating negative nutritional balance. While the non-absorbability during cholera of oral plain saline without glucose was shown in balance studies, such studies were rarely performed in non-cholera pediatric NDDs. Multiple antibiotics were used [84] in the era before rotavirus and other viral enteric pathogens were discovered, despite no benefit for most patients.

Use of oral glucose as part of cholera or NDD treatment continued as a recommendation without provision of objective evidence of caloric or absorptive efficacy or net gut balance studies. Such dietary recommendations, along with many other oral foods or liquids (including oral plain saline without glucose) were unaccompanied by any reproducible method or objective results in terms of reducing or eliminating I.V. fluid requirements or eliminating high CFRs. If vomiting occurred, parenteral infusions were given, often for many days.

There were no descriptions of any therapeutic method of administration linking input to output or reference to any objective evidence of effect on outcomes. Formulaic rules for parenteral therapy in hospitals or rehydration centers were often bewilderingly complex [89].

Powers’ recommendations dominated pediatric NDD therapy over five decades, including the inappropriate use of blood transfusions or plasma, which must have spread untold infections with hepatitis C and B and other blood-borne diseases. Liberal use of plain water by mouth or nasogastric tube during the first 48 h risked hyponatremia, and slowly absorbed, painful I.P. and S.C. infusions were easily infected and disfiguring (especially S.C. 5–10% glucose). Treatment often included prolonged starvation and restriction of oral solutions to the mildest cases or in late maintenance largely as a bridge to resumption of diet. Sugars were often omitted from oral electrolyte solutions without determining absorbability without them. Vomiting was widely regarded as an absolute contraindication to oral intake without determining quantity and clinical importance. Antibiotic use was indiscriminate, though few cases involved susceptible microorganisms (rotavirus and other enteric viral pathogens had not yet been discovered.)

The overwhelming concern about vomiting, effect of oral intake on stool volume and the belief in the ameliatory effect of starvation provided a strong negative bias against expansion of oral rehydration and maintenance therapy during diarrhea. Globally, the devastating effect on nutritional status of repeated episodes of starvation therapy on infants experiencing ten or more diarrhea episodes per year was long ignored.

Darrow’s influential publications [74,75,79,82,88] employed an initial 2–5 day starvation period, but his I.V. and oral potassium balance studies demonstrated the importance of replacing potassium adequately (still an issue today) but did not include studies of complete oral rehydration or maintenance solutions. Inadequacy of potassium replacement persisted, particularly in areas of widespread potassium deficiency, due partly to reaction to deaths from overaggressive potassium infusion [75]. Through the 1950s and 1960s, parenteral fluids were usually continued for 2 days or occasionally for 3–5 days **[90]**. Harrison recommended 12 days of parenteral therapy [84], including blood, plasma and I.V. glucose-electrolyte infusions in severely dehydrated cases. CRFs remained relatively high despite reductions with improved parenteral therapy [88]. Withholding of food and oral fluids for variable periods remained part of routine therapy and continued feeding in early diarrhea was considered irrational [86,90] even after the nutritional benefits of early feeding were demonstrated [80,87]. It was stated that “in most cases diarrhea ceases after suspension of oral intake and start of parenteral therapy” [89].

In patients with mild or absent dehydration, most of short duration, almost any oral fluid intake that does not aggravate diarrhea or electrolyte inbalance is safe to recommend, and continued feeding avoids the harmful effects of starvation therapy. The repeated stress on “resting the stomach”, “N.P.O”, and the like as an essential part of the therapeutic approach to dehydrated pediatric diarrhea patients, and the concern that oral intake would aggravate vomiting and diarrhea placed barriers blocking use of oral rehydration or oral maintenance therapy as initial treatment for patients with profuse dehydrating diarrhea prior to convalescence.

For years after the sugar/sodium transport literature grew, the pediatric literature reflected little or no awareness of the effect of those sugars on intestinal absorption or net gut balance. Sugar concentrations in oral solutions used for caloric purposes were excessively high, leading to complications when absorptive capacity was exceeded [86].

While oral solutions with sugars added for caloric content in convalescent or mild, non-dehydrated patients reportedly began in the U.S. in 1946 [91], oral intake continued to be restricted during early hours of therapy and transfusions of blood, plasma or albumen persisted [90] when dehydration was detected.

In contrast, “oral” (actually almost always nasogastrically administered) solutions were used in South America at least as early as 1943 [93] initially using electrolyte solutions with or *without* sugars, with no balance studies to demonstrate efficacy. As noted above, oral plain electrolyte solutions aggravate cholera, and while not properly studied in pediatric diarrheas before 1968, the same was true in one published case [89]. The rationale in 1943 was partly to avoid the harm resulting from infusions by S.C. and I.P. routes [93].

In the 1950s and 1960s, the use of electrolyte solutions, often but not always with glucose or sucrose added as a source of calories, had wide application in South and Central America [93,94,95,96,97,98,99,100,101] and So. Africa (102–104), administered chiefly by nasogastric tube to infants with relative mild dehydration. Fixed volumes were given without reference to actual volume of losses. Failures were promptly hospitalized; but even with close supervision, CFRs were as high as 6.3%, compared to under 1% using modern rehydration and maintenance therapy, though a significant improvement over prior local interventions. With 24 h coverage at one hydration center, 20.4% were hospitalized and follow-up revealed a 14% CFR after hospital discharge [99]; 77% were successfully treated by monitoring clinical signs, but 7.4% required re-admission.

Various nasogastric or oral solution formulations were used in addition to I.V. therapy [94,95], the oral fluids ranging from water, to tea, Ringer’s or other saline solutions with or without sugar, sugar solutions with low sodium, etc. One formula closely matched one shown effective in modern studies [96,97], albeit using sucrose instead of glucose, sucrose being less effective [98] though usable if glucose is unavailable.

**Figure 1:**
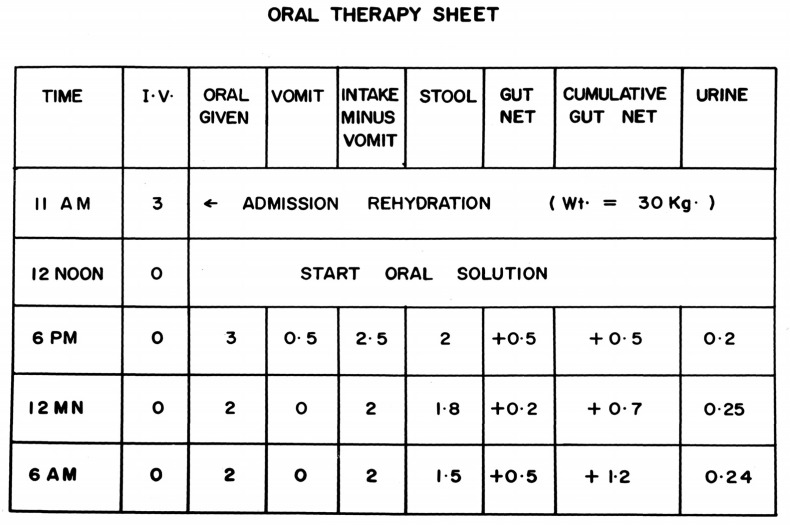
ORT I & O SHEET: figures in liters.

The reliance on gastroclysis necessitated administration in hospitals or special rehydration centers with insufficient staff to cover night and holiday shifts adequately [99]. Patients were intermittently unattended and mothers were on their own for prolonged periods if they were permitted to participate at all. The nasogastric tubes obviated direct maternal control of therapy and required restraining infants by wrapping them in sheets to prevent pulling out nasogastric tubes or turn to a position impeding flow ([99], see Figure 1). Occasional deaths occurred when restrained infants aspirated regurgitated fluids. Complex formulas used to calculate fluid requirements ensured dependence on the medical supervision which was intermittently absent. Actual volumes of diarrheal and vomitus losses usually went unmeasured, nor were intake and output routinely monitored. There was also no published rigorous system of monitoring clinical signs of hydration status on a continuous basis during therapy.

Chlorophenothiazine was often used in attempting to control vomiting as a cause of failure, despite a 23% incidence of significant side effects [100]. Antibiotics were given frequently, and it took years to recognize their lack of benefit in most cases [100]. Gastroclysis was never intended for use in severely dehydrated patients for either rehydration or maintenance fluid and electrolyte therapy and was eclipsed by the rapid development of centers relying on I.V. infusions. The use of oral glucose for calories, along with electrolytes beginning after the acute phase, remained essentially a dietary therapeutic recommendation. Lower CFRs were achieved chiefly at centers employing improved use of I.V. electrolyte therapy. The efficacy of gastroclysis relative to patients’ degree of dehydration and balance data was never objectively established, though high rates of treatments avoiding parenteral fluids were reported from some centers [99,100]. Attempts to transfer therapy to homes were problematic for infants with profuse diarrhea in the absence of clear guidelines for mothers and depended on elaborate personnel and referral facilities needed to ensure success [95].

In the U.S., distribution of high-sugar commercial formulas and maternal errors in preparing safe solutions led to epidemics of hypernatremia [86,91], and later of hyponatremia due to use of low-sodium (30 mEq/L) oral solutions [92]. Hospitalization for even relatively mild dehydration was far more profitable even when outpatient treatment would have sufficed, and the decreased incidence of severe dehydrating diarrhea diminished staff expertise in its management and triage.

In the U.K. [78], Lawson used oral half-strength Hartmann’s solution (no sugar) in the mildest cases, but gave serum I.V. Vomiting was considered an absolute contraindication to oral feeding. CFR was 7%. In South Africa, oral Darrow’s solution was given after S.C. infusions [102]. CFR was 14%. Another center [103] used oral or nasogastric half-Darrow’s solution with glucose for up to 48 h. CFRs were omitted. Bowie [104] reported a regime of half-strength Darrow’s solution in 1.5% dextrose by mouth for 24 h unless vomiting persisted. CFR was, nevertheless, 9.5%.

## 13. Chatterjee

A widely quoted misinterpretation regarding the origins of modern oral rehydration and maintenance therapy arose from H.N. Chatterjee’s 1953 Lancet article [105] titled “Control of vomiting in cholera and oral replacement of fluid.” No balance data were presented to establish that net absorption was occurring in the small number of selected convalescent or mildly ill patients treated orally. He described the use of promethazine and chlorotheophylline (“Avomine”) to treat vomiting in cholera patients. The patients selected for treatment were those whose condition was “relatively satisfactory”. Vomiting stopped after one Avomine tablet in mild cases, and up to six tablets were given to patients whose vomiting persisted 24 h. In modern studies, vomiting stops quickly after rapid rehydration and correction of acidosis in most cases, rarely persisting up to 24 h after admission. So the value of “Avomine” was not in fact established by its uncontrolled use. Chatterjee claimed, however, that Avomine use permitted oral therapy with a hypotonic solution (NaCl 4 g, 25 g glucose and later 2 g KCl per L) by stopping vomiting. Cholera diarrhea, however, continued, for which he gave the juice of crushed leaves of *Coleus aromaticus,* an antidiarrheal folk remedy which is rich in forskolin, a cyclic AMP enhancer [106]. This in theory should aggravate diarrhea as do cholera toxin or VIP, but Chatterjee claimed it controlled the diarrhea, permitting oral therapy. (No controlled clinical trial confirming his claims was performed.) He stated that 33 mild cases among 1093 cholera patients (3%) were successfully treated using this tripartite regimen and an additional 153 moderately severe cases received oral and *rectally* administered glucose-saline solution. Only 17% of the 1093 cholera patients received no I.V. therapy, the majority of his patients receiving only I.V. therapy. As there is no evidence that rectal glucose-saline solutions have any beneficial effect in cholera, his reported success, with no balance data, probably was due to his choice of mildly ill patients rather than his treatment regimen. His solution contained 68 mEa/L Na^+^ vs. 133 in adult cholera stool.

Without balance data, the amount of net absorption of Chatterjee’s oral solution is unknown. His report is anecdotal rather than evidentiary, even for his selected non-severe patients. His choice of glucose was not based on any effect on intestinal absorption, as such data did not appear until 1959 [64].

Separately [107], he reported that antibiotics, including tetracyclines, did not check the diarrhea or reduce mortality in cholera (proof that antibiotics did in fact shorten cholera diarrhea duration and volume appeared later). Therefore, he gave crude juice from *Coleus aromaticus* to 200 cholera patients whose diarrhea stopped within 24 h in 40%, 48 h in 74% and 72 h in 92.5%. Controls (every 6th case) received kaolin and bismuth suspension, and their diarrhea stopped within 24 h in 55%, 48 h in 12.5% and 73 h in 30%. Both groups received routine treatment of shock, presumably with I.V. therapy. Patients also received antihistamines (then believed protective against uremia) and vitamin C. Whether the *Coleus* extract had an antidiarrheal or antibacterial effect remains unknown; Chatterjee noted that it caused an early appearance of rough colonies of *V. cholerae,* whereas smooth colonies persisted in the control group.

## 14. The Translational Steps Leading to Modern Oral Rehydration and Maintenance Therapy

The advance to a successful practical oral rehydration and maintenance therapy has been meticulously recorded by Joshua Ruxin based on tape recordings of all the principle workers involved in its evolution [108]. This documents the essential links between empirical and laboratory basic science and the crucial role of clinical insight and methodology. A brief summary with some additional details will illustrate some parallels between the evolution of I.V. and oral treatments for cholera and NDDs.

In the early 1960s, Phillips and coworkers measured changes in net diarrheal losses during intestinal perfusion of plain saline in cholera patients and found it unabsorbable [109]. This was consistent with the paralyzed sodium pump theory of cholera pathophysiology. Potassium and bicarbonate were found to be absorbable during cholera. He reported that plain water was also absorbable, but this observation was possibly incorrect, based on mistaken interpretation of the finding that the patients given plain water to drink developed a slight increase in urinary output of low sp.gr.

Given the derangement of intestinal osmoregulation by cholera toxin demonstrated in the dog cholera model [110], hypotonic intestinal saline solutions are not absorbable, but their luminal tonicity is adjusted by increased plasma to lumen sodium chloride secretion. In cholera patients, this leads rapidly to marked negative sodium and chloride balance. Resulting hyponatremia triggers ADH suppression and consequent increased hypoosmolar urine output [111] that Phillips took as proof of water absorption. His studies had shown major net sodium losses and rising plasma sp.gr. during intestinal plain water perfusions [109]. Thus, the extent, if any, of plain water absorption during cholera remains an open question. His statement that a patient drinking water was maintained in water balance is contradicted by the rise in plasma sp.gr. during the oral period.

Phillips then added glucose to the perfused saline solution and observed for the first time that the glucose was absorbed and induced a reduction in the net rate of sodium ion losses, indicating that the coupled glucose-saline transport mechanism was not inactivated in cholera [16]. According to several sources [108] and as Phillips stated [16], his choice of glucose to add to plain saline solutions for intestinal perfusion of cholera patients was to determine the intestinal response to raising the saline solution tonicity with a nonelectrolyte. The effect of glucose on water absorption was not mentioned, since he believed that plain water was absorbable by itself in cholera [109].

Phillips’ observations in the early 1960s were made at the very beginning of what afterwards became a vast literature on coupled substrate/sodium absorption, but little had been published at the time, and the early studies were in vitro and in animal models, published in journals unlikely to have been available to NAMRU-2 in Taipei, since in those days journals arrived many months late. His early work omitted those references, suggesting, as he indicated, that his glucose observation was empirical, exploratory, investigational and open ended. After observing the decreased net salt losses accompanying the addition of glucose to the saline perfusions, he proposed a new therapeutic paradigm: to use glucose to ***stop*** the cholera diarrhea by promoting reabsorption of intestinal luminal fluid. The question of the ongoing effects of cholera toxin on the intestinal mucosa was not addressed:

“We also demonstrated that dextrose when given by mouth is absorbed and in its absorption sodium and chloride ions are absorbed along with water, with an amelioration of the diarrhea. This is a dose-dependent response but unfortunately, if sufficient dextrose is given to *stop the diarrhea* (italics mine), most patients develop nausea and vomiting. Thus the hope for a simple method of treating cholera by this procedure did not materialize.” [112]. Translational progress was derailed by a faulty therapeutic paradigm: he believed that glucose could promote absorption to such an extent as to *stop* the cholera diarrhea abruptly; patients would reabsorb their own diarrhea fluid. Intake and output measurements would be obviated, along with the need for medical staff, hospitals and I.V. fluids. To achieve this, he devised a highly concentrated glucose and electrolyte solution to be given to acute phase cholera patients by oral or nasogastric routes.

Though he was later characterized by some as uninterested in practical applications and in pursuit of only basic science goals [108], he was in fact eager to test the glucose finding a potential treatment breakthrough, and sent a team to test the concentrated glucose and saline solution during a cholera outbreak in the Philippines.

The Philippine trial was a failure, and 5 of some 30 patients died. Deaths were attributed by a visiting observer to cardiopulmonary decompensation from overhydration due to combined absorption of the oral or nasogastric solution plus continued excessive I.V. infusions [108]. A likely precipitating factor was massive net water loss into the intestinal lumen precipitated by the hyperosmolar concentrated solution used. The defect in osmoregulation of luminal contents in cholera, in which hypertonic solutions are adjusted chiefly by rapid luminal influx of water [110], was not yet identified.

Ironically, Phillips, whose work led to the modern I.V. therapy of cholera based on balance studies and intake and output measurements, had not conceived of the alternative paradigm of using an isotonic oral glucose-electrolyte solution for replacing ongoing losses of water and electrolyte balance after correcting shock with I.V. rehydration fluids (or rehydrating and maintaining patients not in shock with the oral solution alone).

The most likely reason for this failure was Phillips’ assumption that the solution would stop the diarrhea by causing patients to reabsorb their own rice water fluid [112]. The alternative of having patients drink up to 100 L of an absorbable solution to replace their fluid losses must have been viewed, if at all, as impossible to implement in most cholera-affected areas, which had no medical personnel, supplies and equipment to implement the matching principle. The possible solution of tying the amount of oral solution to be imbibed to the patient’s clinical signs of hydration status was not then considered. Another factor may have been his adherence to the theory of the paralyzed sodium pump theory of cholera pathophysiology [113]: if glucose freed up the paralyzed pump, it might stop the diarrhea. However, glucose was not an antidote in the traditional sense; it could enhance salt and water absorption to replace diarrheal losses, not stop them.

Importantly, the adjuvant value of antibiotics in shortening the duration of cholera had not yet been discovered. Many antibiotic trials had “failed” due to flawed designs in which mortality was the endpoint in studies in which fluid and electrolyte therapy was inadequate. In that setting, the value of adequate antibiotics could not be revealed. Only after the use of tetracycline (and later other appropriate antibiotics) along with rehydration and maintenance I.V. therapy was tested in 1964 could the shortening of diarrhea from a mean of 1.8 down to 0.8 days be revealed [114,115]. Before this beneficial effect of antibiotic therapy on cholera duration was demonstrated, the prospect of up to 9 days of large volume oral therapy probably seemed unachievable.

Phillips’ research strategy was notable for n of 1 studies, which he used effectively when studying basic physiological functions which varied little between subjects. If the result came up positive, a larger confirmatory study could be performed; if negative, further studies would await additional supportive data. Many of his cholera studies were performed on one or two subjects.

In 1965, Love at NAMRU II, using the n of 1 approach, referenced the active transport literature and reported that in the rabbit ileal loop cholera model, net water and salt absorption followed use of a glucose-containing electrolyte solution, noting the apparent contradiction of the sodium pump paralysis concept [116]. He also used the solution orally in a single (relatively mildly ill) cholera patient to demonstrate brief achievement of net positive gut balance. Love mentioned the therapeutic potential, but his report did not lead to renewed interest in a clinical trial based on the new principle, suggesting that the negative aftermath of the failed Philippine clinical trial persisted.

The shock of the failed trial was traumatic enough to bias Phillips against allowing any further attempt to develop an oral therapy for cholera, and there the matter might well have ended had David Sachar not arrived on the scene to serendipitously revive interest in the glucose issue that Phillips had serendipitously discovered.

As Ruxin noted [108], Sachar and Hirschhorn were among the series of young investigators sent abroad to conduct research on diseases which were of military importance. At the time, such assignments at NIH in the Public Health Service satisfied the requirement for military service during the Vietnam war. Sachar’s study in cholera patients would extend Love’s findings in the rabbit model and one cholera patient and would again test Phillips’ theory that cholera resulted from paralysis of “the sodium pump”. He trained to measure intestinal transmural electrical potentials at Ussing’s lab in Denmark, and arrived with an apparatus to do so in cholera patients. When glucose or galactose was added to the luminal saline solution, an increased potential appeared, indicating sodium absorption [117]. Hirschhorn, observing Sachar’s results, recognized the potential importance of this finding beyond the conflict it created with the paralyzed sodium pump theory, and asked Phillips’ permission to study it further. Phillips had, after publishing the brief mention of the glucose effect in 1964 [16], suppressed information about the failed Philippine oral therapy trial and withheld approval, sharing with Hirschhorn the failed Philippine ORT trial as an indication that the oral route would never be safe or effective.

However, Hirschhorn convinced a reluctant Phillips to permit a further study not for any therapeutic goal, but to explore further the absorption of glucose and other sugars in cholera patients. Communications exchanged between the investigators at the PSCRL, Dacca (now Dhaka) and the Johns Hopkins Center for Medical Research and Training (JHCMRT), Calcutta (now Kolkata) led to a study confirming achievement of positive net gut balance during glucose-electrolyte perfusion periods, as Love had shown in a single patient. The link to the transport literature was now firm Hirschhorn was about to return home, as were the lead ICMRT investigators, and the final translational step remained uncertain and unrealized.

At the 1967 U.S.–Japan cholera conference in Los Altos, brief abstracts of the two groups’ findings were presented, stating that: “Glucose and galactose (two sugars associated with the enhanced active transport of sodium) markedly reduce the stool output in actively purging cholera patients when given by mouth along with isotonic electrolyte solution in large quantities” [118].

Actually, it was net diarrhea fluid losses, not stool output per se that was reduced, and the sugar-electrolyte solutions were given not by mouth but solely by nasogastric or intestinal tubing. Net positive gut balance was not actually mentioned.

The ICMRT abstract stated that “Significant absorption of water, glucose and electrolytes was observed which varied with glucose concentration” [119]. It is notable that nothing but these two brief abstracts were published or made available in draft form before the successful spring 1968 oral maintenance trial in Dhaka was complete.

In final published form in July, 1968, the findings were summarized somewhat differently: ”The rate of intestinal fluid loss was decreased significantly….when electrolyte solutions containing glucose were administered intragastrically or into the intestine for periods of 12 to 32 h [120]” and “A study of patients with severe cholera has demonstrated absorption of glucose and a definite improvement in net water and electrolyte balance during intragastric infusion of glucose-electrolyte solution. In most patients studied, water, electrolyte and acid-base balance were maintained satisfactorily for 12 h (out of a total 48 h study period) solely by the intragastric infusion of glucose-electrolyte solution.” [121]. The two reports concluded that “further investigation of the role [of oral solutions] was warranted but cautioned that intravenous solutions remain the mainstay of the successful treatment of cholera” [120], and “The results suggest that oral glucose therapy could be of value in the treatment of cholera and that the requirement for expensive and scarce I.V. fluids may be reduced thereby” [121]. The possibility that the findings might have significance for NDDs beyond cholera was not mentioned.

In sum, the studies of Hirschhorn, Pierce and co-workers confirmed and expanded Phillips’ earlier report of the glucose effect in cholera patients, but did not constitute or demonstrate the efficacy of oral or nasogastric fluids for eliminating the need for intravenous maintenance or rehydration therapy. This time the added linkage to the factor of the “intact” (vide infra) active transport mechanism of sodium, and with it chloride and water, during cholera when glucose was present shifted the therapeutic paradigm from stopping the diarrhea to diminishing the net fluid and electrolyte losses by infusing glucose plus electrolyte solutions via intragastric or intraintestinal tubes. The losses were still huge and prolonged, and it was not clear how this would become a useful practical treatment.

Oral therapy per se did not yet exist, since both Hirschhorn and Pierce and colleagues had kept patients on I.V. fluid drips during the study, and the study periods with gastric or intestinal perfusion via plastic tubes were alternated with lengthy periods of I.V. maintenance therapy. Neither report included the volumes of I.V. fluids the patients received. Patients’ fluid losses were huge and long-lasting, and the problem of vomiting and the methodological context remained unresolved issues. Importantly, Hirschhorn, Pierce and colleagues had shifted away from the therapeutic paradigm of stopping the diarrhea, while illuminating a broader range of responses to sugars in cholera patients. Demonstration of the (clinical bedside) success of oral rehydration or maintenance therapy per se and proofs of efficacy and of effectiveness in the field were still awaited, but without Hirschhorn’s and Pierce’s finding that intragastric glucose-electrolytes infusions improved sodium absorption and net water and salt balance during cholera, Phillips’ finding that glucose did not stop the diarrhea might have kept the oral therapy concept buried as a few lines in the literature, as quoted above.

In recent years, it has been found that substrate-enhanced active sodium transport in cholera is not merely intact as previously thought, but is in fact ***increased*** [122]. This fascinating paradox of increased absorption of sugars (and some amino acids) enhancing active transport of salt and, with it, water during the most profound diarrhea, arising from chloride secretion linked by the cystic fibrosis transmembrane regulator (CFTR), awaits further research in the quest for a therapeutic intervention capable of quickly stopping cholera diarrhea, as sought from O’Shaughnessy’s to Phillips’ time and today.

The fall of 1967 arrived with a unique absence of cholera in Dacca (now Dhaka). PSCRL staff, equipment and supplies were moved to Malumghat, between Chittagong and Cox’s Bazaar to help manage a cholera outbreak near the Christian Memorial Hospital located there. On arrival, we found the wards empty; local mullahs had warned the affected Muslim village populations against entering the Christian hospital, claiming the “sign of the pig” would be put on their foreheads. As cholera was seen as always fatal, they were persuaded to let their beloved family members die in the huts. Dr. Zahidul Haque, a Chittagonian dialect speaker, joined a visit to the affected villages to convince the elders to permit treatment of their affected family members. Ultimately, convincing them necessitated starting I.V.s in the huts, demonstrating, as Latta had described, the apparent miracle of the almost dead rising back to life within minutes. The hospital wards soon filled up [123].

The former Director General of Health of East Pakistan, Dr. Fahimuddin, led a visit to the local thana (police station) to check the scope of the epidemic. The officer in charge was relaxing with his feet crossed up on his desk. As Fahimuddin had a quiet, unassuming manner, the officer did not budge until we informed him who Fahimuddin was, upon which he hastily jumped to his feet and nervously asked how he could assist us. His response to Fahimuddin’s question as to the extent of cholera in the area was to insist that there was none, though we had been treating patients there. This gave immediate insight into the total lack of reporting of cases and the absence of any governmental will, compassion, organizational ability and resources to prevent cholera deaths. The thought of the patients dying unattended in their village huts reinforced the urgency of developing a treatment that would overcome the factors preventing life-saving medical care from reaching them: cost, non-availability of supplies, lack of trained personnel and governmental inaction. It could be available in every village. Additionally, if it worked in cholera, it would work in all the less profuse, albeit lethal NDDs affecting both children and adults.

No publication on the glucose effect in cholera was yet available except Phillips’ original observation and the brief abstracts presented at Palo Alto. However, Rafiqul Islam, a PSCRL staff clinician, had authored a protocol to be implemented at the Malumghat hospital, of the feasibility of a nasogastrically administered glucose solution whose electrolyte composition, though low in potassium, approximated that of cholera diarrhea. The version implemented differed in minor details from the version included in the PSCRL 1967 Technical Committee Report [124]. The method, apparently adopted from that used in Hirschhorn’s study, was flawed in that patients were given fixed quantities of the solution with no matching between volumes of losses and of oral fluid intake. After intravenous fluids corrected shock, patients who lost a liter per hour but were given the fixed volume of 500 or 750 mL of oral solution per hour (depending on weight) became rapidly dehydrated and slipped back towards shock, necessitating termination of the study. Patients who lost 250 mL per hour but received 500–750 mL per hour of oral solution soon became edematous due to overhydration. This was the second oral therapy study to fail, due partly to the idea that only a highly simplified method, requiring only a fixed oral dose with the fewest measurements or clinical skills, could possibly be useful. Even though the annual increases in patient admissions to the cholera hospital’s wards in Dhaka had reached levels challenging the ability of the hospital to meet I.V. fluid production needs, the use of oral maintenance therapy as a means of drastically reducing those needs remained unrealized.

When the trial was terminated, careful review of the data made the pattern of failure evident: underhydration, overhydration, underhydration, overhydration… yielding the insight that oral therapy ***had*** to work if only a method of matching the losses with equal volumes of an oral glucose plus electrolytes solution closely matching the electrolyte composition of cholera diarrhea was used. Being new to cholera and having just mastered basic intake and output monitoring of cholera patients, using Watten cots and I.V. therapy with 5–4-1 solution for both rehydration and maintenance, the crucial oral therapeutic methodology fell into place. A new revised oral maintenance therapy protocol was drafted using a matching volumes method.

Patients either drank the solution or received it or by nasogastric tube: the results were the same, showing that nasogastric tubes were unnecessary. Intravenous needs of the most severely dehydrated patients in shock were reduced by 80%. Subsequent studies showed that most patients with non-cholera diarrheas could be rehydrated and maintained with oral fluids alone, using the new methodology. Vomiting, which had posed a psychological barrier to oral intake, proved to constitute in the majority of patients an insignificant fluid volume, not a barrier to positive fluid balance, at this stage of disease. Future training in ORT technique would focus on this point.

The translational development of an absorbable oral solution and an effective methodology of administering it addressed the persistent unavailability of the intravenous fluids and therapeutic methodology (persisting even today as reflected by the CFRs in some recent cholera and AWD outbreaks). Eight years passed from Phillips’ first observation that cholera patients’ intestine could absorb oral saline if glucose was present to the first translation into an effective therapeutic method was published in the Lancet 17 August 1968 [3]. Further studies proved that most cholera and NDD patients not in shock, and even those with severe dehydration and moderate hypotension [9,66,67,125,126,127] could be rehydrated and maintained using oral glucose-electrolytes solutions alone. Today, over 90% of patients can be rehydrated and maintained with ORT alone. An exception is patients early in the course of severe cholera with early massive vomiting [128].

The development of modern oral glucose-electrolyte rehydration and maintenance therapy as initial treatment, and the completion of treatment within a relatively short period, radically altered therapy and made it possible to extend it beyond hospitals and treatment centers into homes.

The final translational breakthrough was the realization that volume of oral intake had to match volume of losses. Since, with tetracycline, diarrhea volume decreased in each successive 4–6 h monitoring period, matching previous periods’ losses ensured positive gut net water and electrolyte balance and maintenance of hydration (Figure 1).

Treatment monitoring forms were used in the pivotal first successful oral therapy study using an ORS averaging the adult and pediatric cholera stool electrolyte compositions [2]. After correction of shock with initial I.V. rehydration, patient received the oral glucose-saline solution to drink. Diarrhea and vomitus volumes were measured using Watten cots, calibrated stool buckets and bedside basins. Volume of oral solution to drink was matched to volume of losses in the previous 4 or 6 h intake and output (I & O) period. Tetracycline given orally after rehydration ensured duration would average 32 h [114,115], during which gut net balance (oral solution volume imbibed minus diarrheal losses in the bucket) was monitored. (Other antibiotics can be used based on vibrio antimicrobial sensitivities, particularly for children.)

Note that the I & O record shows that matching the volume oF ORS to drink to the volume of losses in the previous I & O period results in sustained positive net gut balance, since cholera diarrhea volume decreases with successive I & O periods when adjuvant antibacterials are given. In most cases, vomitus losses proved negligible compared to diarrhea losses.

In retrospect, this seems an obvious conclusion, already employed for intravenous therapy, but it had escaped a series of investigators skilled in the field, due in part to the magnitude of losses, the exaggerated fear of vomiting compared to actual vomitus losses of patients at this stage of their disease, the hesitation to accept the feasibility of using such a method outside of the hospital orbit, and the bias towards attempts to stop rather than replace the outflow of water and salts. The traditional framework of hospital-based rather than home-based treatment also played a role.

The matching principle employed an ORS formulation containing electrolyte concentrations close to those in cholera stool. Use of lower concentrations requires cholera patients to drink volumes exceeding losses, to avoid electrolyte imbalances, a method that may exceed patients’ drinking capacity and possibly raise risk of overhydration. For use in pediatric NDDs, the 2:1 ratio of oral solution intake to water was successful in many studies, while avoiding (and even treating) clinically significant hypernatremia or hyponatremia [98,125,126,127].

The puzzle of how to successfully extend such a therapy to patients at home and before dehydration became clinically significant depended on a method of using the therapeutic paradigm and effectively adapting it to clinical signs and symptoms of hydration status recognizable by mothers, enabling them to use the ORS successfully at home. It could reach every village. Additionally, if it worked in cholera, it would work in all the less profuse, albeit lethal non-cholera AWDs affecting both children and adults.

A successful large field trial conducted at the rudimentary Matlab Bazaar field treatment center using paramedical workers and nurses [65] set the stage for the use of oral rehydration and maintenance therapy in a epidemic of cholera (79% in one sample of 100 patients) and cholera-like ADWDs under disaster conditions. Indira Gandhi’s office was notified of the potential for the new ORT method to ameliorate the cholera epidemic then in the refugee camps near Kolkata during the 1971 refugee crisis in India precipitated by the Bangladesh independence war, and forwarded the information to the Indian Ministry of Health on or before 21 June 1971 [129]. Shortly afterwards, Dilip Mahalanabis and colleagues arrived and utilized the WHO formulation of ORS (90 mEq Na^+^) to save many cholera patients’ lives in the camps with limited amounts of I.V. fluid available, extending the use of oral maintenance to using family members and paramedicals to keep patients drinking, and oral rehydration alone in milder cases. CFR was 3.6% among 3700 patients treated, compared to an estimated 30% in the camps in general [130].

The success in treating the 3700 refugees (79% confirmed cholera in 100 sampled) helped secure the strong support of the WHO, UNICEF, the USAID to mount a global ORT program with enormous impact. Adaptation of the ORT method was extended to less profuse but still often fatal NDDs in hospitals, field treatment centers and in homes with a strong emphasis on effective maternal instruction and substituting monitoring of clinical signs of hydration status for intake and output measurements [131].

Annual mortality from diarrheal diseases among under-5-year-olds fell from five million to under 500,000 by 2018 [6,7].

## 15. Conclusions

What does the review of the history of development of I.V. and oral rehydration and maintenance therapy tell us about translational medicine? Among the factors blocking translational success were the entrenched erroneous concepts and opinions regarding pathophysiologic and therapeutic paradigms and their undue establishment in the medical literature, which, even in the modern era, has been noted to contain a significant proportion of erroneous information [132].

Lapses in clinical methodology also negated correct paradigms, some of them, in retrospect, seemingly simple and obvious. Just as the need to replace water and electrolyte losses orally with volumes of glucose-electrolyte solutions matching those of the preceding I & O period was missed, so was the necessity for maintenance I.V. fluids to prevent recurrent dehydration and shock in the 19th C. development of I.V. therapy, due probably to the prevalent misconception of cholera as a “three-stage” disease terminating in “collapse”.

Clinical science was ahead of the as yet unborn sciences of microbiology and biomedical engineering, referred to by Howard-Jones as the “interdependence” of the different branches of science. This posed the anti-translational barriers of sepsis and air emboli.

The effect of serendipitous empirical observations such as Phillips’ glucose findings revealed a mechanism allowing intestinal absorption in cholera patients, bringing science to the bedside. However, a faulty therapeutic paradigm interrupted translational progress.

The science of active transport, though not in the historical chain of events leading to Phillips’ initial observation of the glucose effect was nonetheless influential in sustaining support for the rationale of oral rehydration and maintenance therapy, even though the heightening of this effect above normal levels in cholera was not recognized until oral therapy was already widely used.

Active transport research also led directly to testing amino acids [17] and other substrates and optimizing electrolyte and substrate concentrations in the ongoing quest for an ORS capable of reducing duration and volume of diarrhea.

Realization of the social and societal consequences of centuries of neglect of the population at risk played a role in the prioritization of the effort to develop an inexpensive and readily available oral therapy, and helped overcome the relatively low priority given applied medicine relative to basic science in the research establishment.

Lastly, the completion of the revolution in diarrhea therapy was the result of translational “side effects”, including abandonment of traditionally recommended but unnecessary blood and blood product transfusions, reduced harm associated with I.V. therapy (multiple venepunctures, need for restraints, overhydration with edema, infection and thromboemboli) and the transfer from hospital/treatment center to home therapy. Additionally the traditional use of oral electrolytes without substrates and of high calorie-oriented glucose or sucrose concentrations prolonging diarrhea and electrolyte imbalance was phased out.

## 16. Afterword

The decimation of annual under 5 AWD deaths from 5 million to approximately half a million by 2020 [6,7] was achieved with the original WHO Oralyte formulation containing 90 mEq/L of sodium. The Oralyte formulation was adaptable for treatment of both cholera and NDD patients, both adults and children, the former when patients drank one and one half times their fluid losses and the latter matching losses using the 2:1 method [9].

Regretfully, the excellent global track record of highly satisfactory effectiveness and safety has undergone retro-translational alterations, and the historical tendency toward “innovations” as “improvements” has resulted in altering the original WHO ORS formulation to a “low” sodium formulation inadequate to maintain sodium balance in cholera patients, in whom the “low-sodium ORS” confers no benefit but has potential for harm from sodium depletion [9]. Its safety in cholera and NDDs has been inadequately studied [9]. Where antibiotic-resistant *V. cholerae* are prevalent, diarrhea persists for 9–10 days and patients receiving oral or nasogastric replacement with the low-sodium ORS will develop severe and life-threatening sodium losses. The low-sodium ORS for cholera and severe NDDs puts at risk the global translational benefits achieved for both cholera and pediatric severe NDDs with the original single Oralyte ORS formulation. A more rational modification, if one were deemed necessary, would be to promote two different ORS formulations, one for cholera and one for NDDs.
